# Mitigating Effect of Psychological Capital on Employees’ Withdrawal Behavior in the Presence of Job Attitudes: Evidence From Five-Star Hotels in Malaysia

**DOI:** 10.3389/fpsyg.2021.617023

**Published:** 2021-03-18

**Authors:** Zhen Yan, Zuraina D. Mansor, Wei C. Choo, Abdul R. Abdullah

**Affiliations:** ^1^Faculty of Hotel Management, Qingdao Vocational and Technical College of Hotel Management, Qingdao, China; ^2^School of Business and Economics, Universiti Putra Malaysia, Serdang, Malaysia

**Keywords:** psychological capital, job attitudes, turnover intention, hotel customer-contact employees, Malaysia

## Abstract

High turnover rate is one of the striking features of the hotel industry and one of the most significant challenges. High turnover rate causes substantial costs for recruitment, selection and training in hotels, on the other hand, it also leads to negative consequences such as the decline of organizational performance and service quality. Thus, it is necessary to search for the root causes of turnover and put forward solutions. This study was designed to examine the impact of psychological capital (PsyCap), organizational commitment (OC), and job satisfaction (JS) on turnover intention among hotel employees. Additionally, it aimed to test the mediating roles of job satisfaction (JS) and organizational commitment (OC). The data were obtained from 228 hotel customer-contact employees with a time lag of two weeks in three waves in Kuala Lumpur based on convenience sampling. A series of structural equation modeling analyses were utilized to investigate hypothesized relationships. The results reveal that there exists a significant and negative impact of PsyCap on employees’ turnover intention and this correlation is partially mediated through two job attitudes. That is to say, to retain hotel talents, five-star hotel management should take proper measures to help employees obtain and maintain positive psychological resources such as PsyCap, on the other hand, how to cultivate positive job attitudes and strengthen their sense of identification and belonging for their organizations is supposed to be more focused on.

## Introduction

Hotel employees’ turnover intention leads to many tangible and intangible negative outcomes. To be specific, high turnover rate has a seriously negative impact on service quality and customer satisfaction (Holtom and Burch, 2015; Kashif et al., 2017), thereby resulting in tarnished brand image and decreased customer loyalty (Dusek et al., 2014). Taking a novel approach, this study draws from both positive psychology and positive organizational behavior to examine whether the core construct of psychological capital (Luthans et al., 2007b) may be a key factor in better understanding not only how to make employees are more satisfied with and committed to their organizations, but also the direct and indirect causes for employees’ turnover. PsyCap is an important personal resource which enhances employees’ ability for development and usefulness at work (e.g., Siu et al., 2015; Grover et al., 2018). PsyCap has been found to be beneficial to both organizations and employees in facilitating desirable work-related outcomes, such as job satisfaction (Olaniyan and Hystad, 2016), work engagement (Guido et al., 2018) and organizational citizenship behavior (Pradhan et al., 2016). PsyCap also mitigates undesirable outcomes such as workplace bullying (Spence Laschinger and Nosko, 2015), counter-productive behaviors (Avey et al., 2011) and turnover intention (Karatepe and Avci, 2017).

However, so far, only a few empirical studies have focused on PsyCap in existing hospitality research. For example, Karatepe and Karadas’s (2014) research indicated that PsyCap could alleviate the work-family conflicts effectively as well as absence and turnover intentions among frontline staff in international five-and four-star chain hotels in Romania. According to Paek et al.’s (2015) study, it was shown that work engagement played a partial mediating role in the effect of PsyCap on affective organizational commitment and job satisfaction. Tsaur et al.’s (2019) investigation revealed that workplace fun could affect PsyCap significantly and positively and PsyCap could partially mediate the influence of workplace fun on work engagement. After reviewing the relating theoretical and empirical literature, this study attempts to fill in various research gaps, which in turn contributes to understanding how PsyCap influences turnover intention with the mediating role of job attitudes in hotel sector.

Firstly, in order to be sustainable and successful, a hotel is not only supposed to evaluate external types of capitals, such as social capital, human capital and cultural capital but also have to assess another two important aspects, namely employees’ PsyCap and attitudinal strengths (Luthans et al., 2008). Additionally, Guan et al. (2014) argue that most of hotel managers attempt to lower employees’ turnover rate by improving incomes, fringe benefits, and working environment. Nevertheless, such focus overlooks the importance of the internal demand of employees as social beings. Therefore, we conducted this study to verify the alleviating effect of psychological determinant on hotel employees’ turnover intention.

Secondly, empirical research pertaining to PsyCap in the existing hospitality knowledge is still in its infancy (Avey, 2014). In consideration of this gap, this study attempts to investigate the joint effects of four indicators of PsyCap on hotel frontline employees’ turnover intention in Malaysia. Turnover has serious consequences for service organizations, including difficulties in finding replacement, recruitment, selection, training, socialized costs and customer perceptions of service quality (Hup et al., 2018). Therefore, how to solve the high rate of turnover is a very practical and challenging problem for hotel managers. Nevertheless, empirical research on the relationship between PsyCap and turnover intention in hotel sector is still scarce (Kim et al., 2017).

Thirdly, JS and OC have been regarded as two crucial job attitudes in the organizational behavior (Tharikh et al., 2016). In reviewing the literature, many scholars have investigated the direct relationship between PsyCap and job attitudes and also the direct relationship between job attitudes and turnover intention (e.g., Karatepe and Avci, 2017; Rajabi et al., 2019). Nevertheless, to our knowledge, the mediating effect of JS and OC on the relationship between PsyCap and turnover intention has not been examined before especially in hospitality industry.

Fourthly, most of the research on PsyCap in services industry has been conducted by Karatepe and his colleagues in Cyprus (e.g., Karatepe and Avci, 2017), Russia (e.g., Ozturk and Karatepe, 2018), and other European countries (e.g., Karatepe and Karadas, 2014). However, there have been relatively few empirical studies on PsyCap in Southeast Asia. We believe that our research will address this gap, as it can provide a rare opportunity to verify the validity and applicability of concepts which have been largely developed in Western cultures.

Grounded in this backdrop, this study aims to examine the joint effects of four indicators of PsyCap on customer-contact employees’ turnover intention through the mediating role of job attitudes in hotel sector in Malaysia.

## Literature Review

### COR Theory and PsyCap

According to Hobfoll’s (1989) conservation of resources (COR) theory, individuals are eager to obtain, preserve and maintain their limited resources. When individuals perceive that their resources are threatened or the return of their formerly invested resources can’t meet their expectations, they will have a sense of insecurity and tend to quit their positions in order to obtain new resources. Hobfoll (2002) further points out that the basic resources of employees in work can be divided into two categories, namely individual resources and relationship resources. Individual resources consist of self-efficacy, self-esteem optimism and so on, which stimulates workers to spare no efforts to achieve their targets when they encounter tough challenges or adversity. In other words, individual resources can make employees keep positive emotions and satisfaction for their jobs and prompt their intrinsic motivation in order to reduce their withdrawal behaviors. In this study, a type of individual resource that is called psychological capital will be more focused on.

Hobfoll (2001) also puts forward that resources have a tendency to generate each other since an individual may have a major type of resource, which is related to or can replace other resources. For example, when a role demands, individual resources such as self-efficacy or resilience will be linked to or replace conditional resources such as person-organization fit or person-group fit. Hobfoll (2001) defines such linkages as resource caravans and believes that resource caravans can result in positive emotions and attitudes.

Luthans and Youssef (2004) originally conceptualized PsyCap and extended it to the field of human resource management. It is a core psychological resource to promote personal growth and performance improvement. The so-called PsyCap refers to a positive psychological state of individuals, which is manifested by self-efficacy, hope, resilience and optimism. As conceptualized by Luthans et al. (2007a), “…(1) having confidence (self-efficacy) to take on and put in the necessary effort to succeed at challenging tasks; (2) making a positive attribution (optimism) about succeeding now and in the future; (3) persevering toward goals, and when necessary, redirecting paths to goals (hope) in order to succeed; and (4) when beset by problems and adversity, sustaining and bouncing back and even beyond (resiliency) to attain success.”

In addition, past studies have investigated the relationship between PsyCap and various employee attitudes and suggested PsyCap is positively related to desirable employee attitudes and negatively related to undesirable employee attitudes (Youssef and Luthans, 2007). For example, PsyCap has been verified to be positively associated with JS and OC among managers in petro-chemical industry in Saudi Arabian (Idris and Manganaro, 2017) and PsyCap can significantly and positively influence JS in hotel sector (Hyo and Hye, 2015).

### Job Satisfaction (JS) and Organizational Commitment (OC)

Researchers in the field of management and organizational behavior have regarded JS and OC as two major job attitudes in the organization (Tharikh et al., 2016).

#### Job Satisfaction (JS)

According to Locke (1969), JS has been conceptualized as individual’s pleasurable emotional state generated by the evaluation of his or her job when achieving the value of job. The significance of JS has been highlighted largely in prior studies in hotel field, because of its positive effect on OC (Bai et al., 2006; Gunlu et al., 2010), organizational performance (Gu and Sen, 2009), retention (Ashton, 2018). Normally, JS can be categorized into two dimensions, namely intrinsic satisfaction and extrinsic satisfaction. Intrinsic satisfaction derives from the characteristics of job itself (e.g., job content, responsibilities, autonomy, variety, achievements), whereas extrinsic satisfaction comes from the context in which the job is performed (e.g., company policies, salaries, fringe benefits, relationships with colleagues and supervisors, job security) (Herzberg et al., 1959).

#### Organizational Commitment (OC)

Organizational commitment has been defined as a firm belief and resolute acceptance of organizational targets and values (Porter et al., 1974), as well as a strong will to work on behalf of their organizations, and also a burning desire to maintain one’s organizational membership. Meyer and Allen (1991) suggest that OC is a three-component conceptualization:

(1) Affective commitment refers to affective attachment to the organization;
(2) Normative commitment is an obligation to retain with the organization;
(3) Continuance commitment refers to the perceived cost associated with leaving the organization.

### Turnover Intention

Turnover intention is defined when employees plan to leave their organizations (Porter et al., 1974). Turnover is inevitable in every organization, with some employees leaving voluntarily and others being fired. Both voluntary and involuntary turnover has serious consequences for organizations, including difficulties in finding replacement, recruitment, selection, training, socialized costs and customer perceptions of service quality (Hup et al., 2018). Many researchers also suggested that turnover intention was the best predictor of an employee’s actual turnover (Griffeth, 2000).

## Hypotheses Development and Research Model

### Direct Effects

A main explanation mechanism for PsyCap’s effect on job attitudes is that those employees who are higher in PsyCap believe that (a) good things will happen at work (optimism), (b) they can achieve success (hope and efficacy) and (c) being able to recover from change (resilience) (Avey et al., 2011). In consideration of the general expectancy of success derived from optimism and the belief in personal abilities derived from efficacy, those high in PsyCap are proved to be more committed to their organizations (Luthans et al., 2008) and more satisfied with their job (Luthans et al., 2007a).

From the perspective of COR theory, PsyCap is personal resource that enable employees to adapt to adversity, cope with difficulties, and perform well in the workplace (Kim et al., 2017). This is because employees high on these resources are thought of having “resource caravans” and such “resource caravans” can lead to positive outcomes (Paek et al., 2015).

After an overview of the recent literature, PsyCap has been found to be closely associated with employees’ job attitudes. Wu and Nguyen (2019) conducted a meta-analysis regarding the relationship between PsyCap and desirable job attitudes. They found significant and large correlations between PsyCap, JS (*r* = 0.51) and OC (*r* = 0.42). Miao et al. (2020) examined the relationship between PsyCap, job attitudes, high-performance work system and interactional justice climate. The results indicated that there existed significant positive relationships between PsyCap with JS (β = 0.44, *p* < 0.01) and affective OC (β = 0.39, *p* < 0.01). In a similar vein, Wirawan et al. (2020) found that the impact of PsyCap on an employee’s JS was significant and positive, with a regression coefficient of (β = 0.32, *p* < 0.01). More literature in hospitality industry also proved that PsyCap was a predictive construct of job attitudes (e.g., Cheung et al., 2019; Viseu et al., 2020; Ribeiro et al., 2021). Thus, based on the above, the following hypotheses are put forward:

**H1:** Psychological capital can significantly and positively influence organizational commitment among customer-contact employees in five-star hotels in Malaysia.
**H2:** Psychological capital can significantly and positively influence job satisfaction among customer-contact employees in five-star hotels in Malaysia.

According to Luthans and Jensen (2005), employees who have higher levels of PsyCap are inclined to stick with their jobs rather than quit. In addition, staff who are able to avail themselves of hope, optimism, resilience and self-efficacy are more confident in handling difficulties at work and tend to stay with their organizations (Karatepe and Karadas, 2014). To be specific, higher levels of optimism about their future and confidence in their ability to succeed in current job can motivate them to take control of their own destinies (Avey et al., 2008), exert substantial efforts and invest resources, and persevere in the face of obstacles (Luthans and Youssef, 2004), rather than become quitters. Additionally, due to higher levels of resilience, even when employees experience negative events in the workplace, those with higher PsyCap are more likely to positively adapt and bounce back from those events, preventing the escalation and development of intentions to quit. Finally, employees with the hope capacity are more able to be successful in their present job in multiple pathways, further reducing the perceived need to leave their organizations.

More recent studies have revealed that PsyCap could effectively mitigate employees’ turnover intention (Brockway, 2019; Seo and Chung, 2019; Sepeng et al., 2020) especially in hospitality industry (Gupta and Shaheen, 2017; Kim et al., 2017; Ozturk and Karatepe, 2018). Accordingly, it is hypothesized that:

**H3:** Psychological Capital can significantly and negatively influence turnover intention among customer-contact employees in five-star hotels in Malaysia.

Past studies have shown that JS and OC are two core mechanisms relating to turnover. For example, in several classic turnover models such as March and Simon (1958) Model, Mobley (1977) Model and Steers and Mowday (1981) Model, these two job attitudes are considered as key antecedents of turnover intention. Albalawi et al. (2019) examined OC, perceived organizational support and perceived alternative job opportunity in predicting employees’ turnover intention in Jordanian SMEs. Finally, they reported that OC had a significant and negative direct effect on turnover intention (β = −0.38, ρ < 0.000). According to Koo et al. (2020), the influence of JS on turnover intention was significant (β = −0.48, *p* < 0.001) among employees in five-star hotels in Seoul. In a similar vein, Hup et al. (2018) revealed that OC and JS were significantly and negatively associated with turnover intention among employees in casino hotels in Macau. Thus, Hypotheses 3 and 4 are proposed:

**H4:** Organizational commitment can significantly and negatively influence turnover intention among customer-contact employees in five-star hotels in Malaysia.
**H5:** Job satisfaction can significantly and negatively influence turnover intention among customer-contact employees in five-star hotels in Malaysia.

### Mediating Effects

Sweetman and Luthans (2010) define PsyCap as a resource that increases positive job-related outcomes and mitigates negative outcomes. According to COR theory, individuals can use their personal resources (e.g., self-efficacy, optimism) to cope with stressful and demanding situations (Hobfoll, 1989) and have favorable outcomes (Hobfoll, 2002). Employees high in PsyCap can handle problems arising from both work and family domains. Such employees also display higher positive job attitudes and lower turnover intention (Abbas et al., 2014). Adequate studies have related PsyCap to job attitudes and turnover intention (Seo and Chung, 2019; Koo et al., 2020; Viseu et al., 2020; Ribeiro et al., 2021). Hence, based on the discussion presented above of the positive effect of PsyCap on employees’ job attitudes and negative effect of job attitudes on turnover intention in a mainstream management context, the following hypotheses are put forward:

**H6:** Organizational commitment can significantly mediate the effect of psychological capital on turnover intention among customer-contact employees in five-star hotels in Malaysia.
**H7:** Job satisfaction can significantly mediate the effect of psychological capital on turnover intention among customer-contact employees in five-star hotels in Malaysia.

### Research Model

**FIGURE 1:**
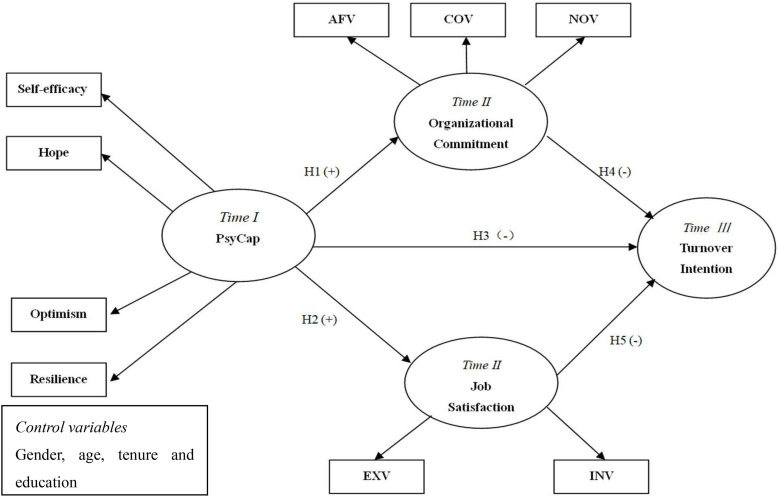
Research model.

The research model shown in Figure 1 demonstrates the hypothesized relationships. To be specific, the model puts forward that PsyCap which is manifested by hope, self-efficacy, optimism, and resilience promotes job attitudes but reduces turnover intention. According to the model, job attitudes mitigate turnover intention. The model also proposes that PsyCap affects the aforementioned job outcome indirectly via job attitudes. Consistent with previous studies (e.g., Griffeth et al., 2000; Hossam, 2014), gender, age, tenure and educational background are controlled in order to avoid potential confounding effects on study variables (VanderWeele and Vansteelandt, 2009; Valente et al., 2017).

## Research Methodology

### Sample and Data Collection

Data were obtained from full-time customer-contact employees (e.g., waiters and waitresses, concierges, guest relations representatives, front desk agents) in five-star hotels in Malaysia. The location of sampling is in Kuala Lumpur since it is the capital city and also one of the leading tourist destinations in Southeast Asia.

According to the statistics obtained from Ministry of Tourism, Arts and Culture Malaysia’s web page at the time of this study, there were totally 29 five-star hotels in Kuala Lumpur (MOTAC, 2020). Convenience sampling technique was utilized to select the hotels to be included in the sample. Finally, management of eight five-star hotels agreed to participate in the study after researchers had contacted the abovementioned hotels.

In current study, because of the constraints on time and money, we utilized the sequential design instead of a fully longitudinal design which could include autoregressive effects (Mitchell and Maxwell, 2013). Thus, data were gathered with a time lag of 2 weeks in three waves (Podsakoff et al., 2012). A list containing customer-contact employees’ names was prepared and a special identity code was given to each respondent. While doing this, researchers attached great importance to confidentiality and anonymity. This code was marked on a hidden place of each questionnaire so that the questionnaires could be matched with each other in three waves.

Respondents who took part in the first-wave investigation (Time I) were also requested to participate in the second- (Time II) and third- (Time III) waves. The researchers would give small gifts to respondents when the questionnaires were completed at every stage. All customer-contact employees were asked to self-administer the questionnaires and return them to their department managers in sealed envelopes. Then all questionnaires were fetched by researchers. The researchers distributed 320 questionnaires in Time I, 273 questionnaires in Time II and 251 questionnaires in Time III. At last, 237 questionnaires were returned. After checking outliers via the Mahalanobis distance (de Maesschalck et al., 2000), five questionnaires were removed. The final sample included 228 customer-contact employees.

Podsakoff et al. (2003) argue that common method bias is a potential risk to the validity of the conclusions regarding relationships among the constructs and can result in measurement error. Consistent with Paek et al.’s (2015) recommendation, current research utilized two procedural remedies to minimize common method bias. Firstly, all respondents were told that participation in the study was voluntary and they were guaranteed confidentiality and anonymity. Second, data pertaining to the predictor (PsyCap; Time I), mediator 1 (JS; Time II), mediator 2 (OC; Time II) and criterion (TI; Time III) variables were collected via a time lag of 2 weeks in three waves.

### Instruments Developments

Validity and reliability are the most important criteria for adopting some specific measurement tools. Additionally, if these tools have been utilized in hospitality studies, they will be selected first. Based on the following four measurement tools, totally 65 items, measuring 10 first-order underlying variables were included in the initial model.

First, a tool of 24-item proposed by Luthans et al. (2007a) has been used to appraise PsyCap. This scale consists of four dimensions and each of them is operationalized with six items. Responses are recorded on Likert five-point scale. Second, according to Allen and Meyer (1990), the scale of OC has three dimensions and eighteen items (three negatively worded items). The validity and reliability of this instrument has been verified iteratively by many hospitality research (Ozturk et al., 2014; Kalidass and Bahron, 2015; López-Cabarcos et al., 2015). Thirdly, the measurement of JS was derived from Weiss et al.’s (1967) Minnesota studies on Vocational Rehabilitation. This questionnaire with high level of reliability and validity has been widely used in hotel-related research (Yurcu and Akinci, 2017; Glaveli et al., 2019). Lastly, for turnover intention, a scale of three items has been utilized. Turnover intention was reliably measured by several shorter scales of 3–5 items in many hotel empirical studies (Zopiatis et al., 2014).

### Analysis Methods

SPSS 23.0 and AMOS 23.0 were employed to analyze the collected data. Firstly, SPSS 23.0 was used to conduct the preliminary descriptive data analysis, and secondly, AMOS 23.0 was utilized to conduct SEM to verify the foregoing hypotheses (Anderson and Gerbing, 1988). On the one hand, a full measurement model was examined via confirmatory factor analysis (CFA). And then, on the other hand, a structural model was analyzed to evaluate posited research hypotheses (Clapp-Smith et al., 2009).

## Results

### Profile of the Sample

Totally three hundred and eighty (*n* = 228) questionnaires were completed and collected. Table 1 demonstrates the demographic characteristics of the respondents in terms of gender, age, tenure, position and educational background.

**TABLE 1:** Profile of the respondents (*n* = 228).

**Characteristics**	**Frequency (*n*)**	**Percentage (%)**
**Gender**		
Male	95	41.7
Female	133	58.3
**Age (years)**		
Under 20	19	8.3
21–30	105	46.1
31–40	66	28.9
41 or older	38	16.7
**Tenure**		
Less than 1 year	78	34.2
1–3 years	93	40.8
4–6 years	28	12.3
More than 6 years	29	12.7
**Department**		
Front office	128	56.2
Food and beverage	100	43.8
**Education**		
SPM	75	32.9
Certificate	25	11.0
Diploma	86	37.7
Bachelor degree and above	42	18.4

### Measurement Model Estimation

The full measurement model which consists of 10 first-order underlying factors and 65 items (respectively optimism: 6; self-efficacy: 6; hope: 6; resilience: 6; intrinsic satisfaction: 12; extrinsic satisfaction: 8; affective commitment: 6; continuance commitment: 6; normative commitment: 6; turnover intention: 3) was assessed to identify the dimensionality, convergent and discriminant validity. Thus, a careful inspection of the CFA results implied that it was necessary to delete three items due to their low factor loading. After three items (HO4, OP1, and RE5) were deleted from the initial full measurement model, the revised full measurement model presented satisfactory fit with the data.

As can be seen in Table 2, all the constructs’ Cronbach alpha coefficients are larger than 0.70 (Hair et al., 1998), which implied that the rest of items could reflect corresponding constructs well. In addition, all standardized factor loadings in the table range from 0.618 to 0.843, which means that all items can measure their corresponding constructs effectively. What’s more, all the constructs’ composite reliability are larger than the minimum threshold of 0.7. According to Fornell and Larcker (1981), under the circumstances, items of each construct were internally reliable and consistent. Finally, AVE values were higher than the threshold value of 0.50, which indicated at least 51.35% of the variance observed in the items could be accounted for by their hypothesized constructs (Fornell and Larcker, 1981). The above findings also indicated that all the items measuring constructs were one-dimensional in the full measurement model.

**TABLE 2:** Results of reliability and convergent validity of full measurement model.

**Variables and items**	**Std. factor loading**	***T*-Value**	***P*-Value**	**C.R.**	**AVE**	**Alpha**
PsyCap: Self-efficacy				0.8783	0.5476	0.877
SE1.	0.759	–				
SE2.	0.691	10.360	***			
SE3.	0.656	9.788	***			
SE4.	0.723	10.877	***			
SE5.	0.767	11.623	***			
SE6.	0.831	12.662	***			
PsyCap: Hope				0.8333	0.5019	0.832
HO1.	0.747	–				
HO2.	0.647	9.183	***			
HO3.	0.643	9.122	***			
HO5.	0.690	9.793	***			
HO6.	0.802	11.290	***			
PsyCap: Optimism				0.8440	0.5202	0.843
OP2.	0.742	–				
OP3.	0.687	9.764	***			
OP4.	0.671	9.527	***			
OP5.	0.738	10.482	***			
OP6.	0.764	10.837	***			
PsyCap: Resilience				0.8609	0.5545	0.858
RE1.	0.745	–				
RE2.	0.720	10.513	***			
RE3.	0.679	9.891	***			
RE4.	0.724	10.572	***			
RE6.	0.845	12.327	***			
OC: Affective				0.8777	0.5469	0.878
AF1.	0.794	–				
AF2.	0.747	11.814	***			
AF3.	0.743	11.737	***			
AF4.	0.601	9.156	***			
AF5.	0.710	11.119	***			
AF6.	0.822	13.260	***			
OC: Continuance				0.8687	0.5251	0.868
CO1.	0.737	–				
CO2.	0.678	9.786	***			
CO3.	0.676	9.766	***			
CO4.	0.713	10.306	***			
CO5.	0.741	10.723	***			
CO6.	0.796	11.527	***			
OC: Normative				0.8634	0.5166	0.860
NO1	0.616					
NO2.	0.748	9.020	***			
NO3.	0.806	9.489	***			
NO4.	0.735	8.904	***			
NO5.	0.798	9.424	***			
NO6	0.578	7.407	***			
JS: Intrinsic				0.9360	0.5498	0.930
IN1.	0.740	–				
IN2.	0.770	12.505	***			
IN3.	0.754	12.205	***			
IN4.	0.733	11.779	***			
IN5.	0.719	11.507	***			
IN6.	0.740	11.911	***			
IN7.	0.674	10.659	***			
IN8.	0.731	11.753	***			
IN9.	0.749	12.089	***			
IN10.	0.757	12.261	***			
IN11.	0.800	13.116	***			
IN12.	0.724	11.612	***			
JS: Extrinsic				0.9084	0.5538	0.914
EX1.	0.712	–				
EX2.	0.733	11.320	***			
EX3.	0.771	11.994	***			
EX4.	0.701	10.760	***			
EX5.	0.723	11.134	***			
EX6.	0.761	11.803	***			
EX7.	0.762	11.835	***			
EX8.	0.786	12.264	***			
TI.				0.8196	0.6028	0.811
TI1.	0.808	–				
TI2.	0.738	11.571	***			
TI3.	0.761	11.992	***			

***P < 0.001. SE, self efficacy; HO, hope; OP, optimism; RE, resilience; AF, affective commitment; CO, continuance commitment; NO, Normative commitment; IN, intrinsic satisfaction; EX, extrinsic satisfaction; TI, turnover intention.

As shown in Table 3, fit indices strongly support the hypothesized full measurement model, for instance, *X*^2^ = 2093.212, DF = 1815; Normed *X*^2^ = 1.153; RMSEA = 0.026; CFI = 0.962; TLI = 0.960, and AIC = 2369.212.

**TABLE 3:** Result of full measurement model analysis.

**CMIN**	**DF**	**CMIN/DF**	**RMSEA**	**CFI**	**IFI**	**TLI**	**AIC**
2093.212	1815	1.153	0.026	0.962	0.962	0.960	2369.212

*GFI, goodness of fit index; AGFI, adjusted goodness of fit index; RMSEA, root mean square error of approximation; NFI, normed fit index; CFI, comparative fit index; IFI, incremental fit index; TLI, Tucker–Lewis index; AIC, Akaike information criterion.*

In this research, discriminant validity was evaluated via the following method. In brief, if all the values of AVE were larger than the corresponding squared correlation coefficients, the discriminant validity of the measurement model could be achieved. This method is deemed as the most rigorous test of discriminant validity. As shown in Table 4, all constructs’ squared correlation coefficients are smaller than the corresponding AVE values, thus a strong proof of the discriminant validity for all constructs is provided.

**TABLE 4:** Descriptive statistics, correlations of variables and the results of discriminant validity test.

	**1**	**2**	**3**	**4**	**5**	**6**	**7**	**8**	**9**	**10**	**11**	**12**	**13**	**14**
1. Gender	–													
2. Age	0.102	–												
3. Tenure	−0.038	0.214**	–											
4. Education	−0.061	0.307**	0.382**	–										
5. SE	0.091	0.047	−0.010	−0.034	***0.740***									
6. HO	−0.073	0.089	−0.031	−0.008	0.488**	***0.708***								
7. OP	0.036	0.077	−0.057	−0.098	0.510**	0.454**	***0.721***							
8. RE	0.034	0.133*	0.022	0.052	0.498**	0.486**	0.554**	***0.745***						
9. AF	−0.072	0.071	−0.049	0.055	0.170*	0.174**	0.123	0.126	0.740					
10. CO	−0.039	0.035	−0.120	0.025	0.115	0.131*	0.042	0.027	0.491**	***0.717***				
11. NO	0.017	0.159*	0.025	0.054	0.192**	0.093	0.171**	0.169*	0.487**	0.576**	***0.725***			
12. IN	0.042	0.044	0.054	0.000	0.206**	0.106	0.132*	0.133*	0.241**	0.326**	0.387**	***0.741***		
13. EX	−0.017	0.113	0.000	−0.022	0.373**	0.309**	0.342**	0.291**	0.300**	0.332**	404**	0.575**	***0.731***	
14. TI	−0.027	−0.123	0.007	−0.036	−0.463**	−0.422**	−0.389**	−0.376**	−0.451**	−0.424**	−0.550**	−0.477**	−0.628**	0.776
Mean	1.54	2.35	2.04	2.42	3.64	3.37	3.86	3.55	3.48	3.59	3.66	3.77	3.47	2.51
S.D.	0.500	0.796	0.685	0.799	0.979	0.959	0.951	0.862	1.02	0.898	0.943	0.857	0.884	0.973

The square root of AVE for each variable is shown with bold italics. SE, self efficacy; OP, optimism; RE, resilience; HO, hope; AF, affective commitment; NO, normative commitment; CO, continuance commitment; IN, intrinsic satisfaction; EX, extrinsic satisfaction; TI, turnover intention. **p < 0.01, *p < 0.05.

### Structural Model Estimation and Hypotheses Testing

**FIGURE 2:**
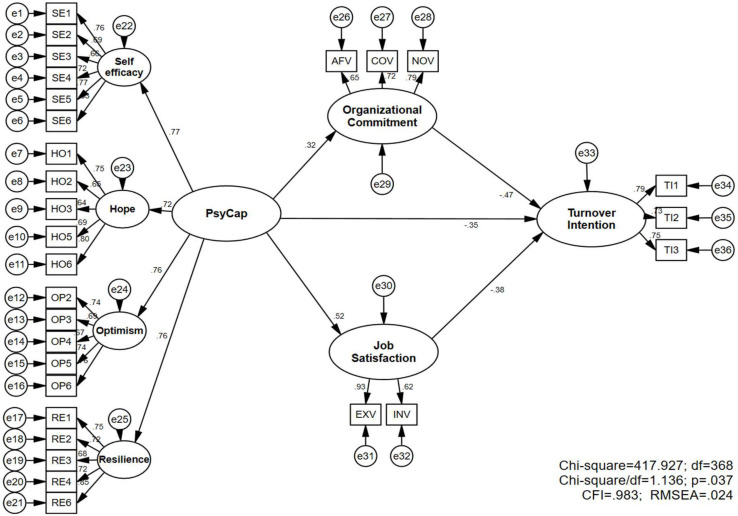
Final structural model.

As the reliability and validity of full measurement model had been verified, the structural model was going to be evaluated and the hypotheses were going to be tested in the next step. After statistical analysis, fit indices from the following structural model (Figure 2) demonstrate that the proposed structural model is satisfactory. In addition, in order to reduce the complexity of structural model, item parceling technique was utilized for two second-order attitudinal constructs.

#### Direct Effects and Hypotheses Testing

Figure 2 shows that there exists a significant and positive correlation between PsyCap and OC (β = 0.320, *p* < 0.001), which supports Hypothesis 1. Besides, there is a significant and positive correlation between PsyCap and JS (β = 0.523, *p* < 0.001), which supports Hypothesis 2. In addition, PsyCap (β = −0.352, *p* < 0.001), OC (β = −0.473, *p* < 0.001) and JS (β = −0.384, *p* < 0.001) all have significant and negative impact on TI, which supports Hypotheses 3, 4, 5.

Tenure was negatively related to JS (β = −0.165, *t* = −3.451) and age was also negatively associated with JS (β = −0.160, *t* = −3.333). Besides, education was negatively related to TI (β = −0.180, *t* = −3.515). The proposed model accounted for 49.8 percent of the variance in job satisfaction, 44.6 percent of variance in OC and 75.3 percent of that in TI.

#### Mediating Effects and Hypotheses Testing

Bootstrapping estimate has been employed to further identify the mediating role of OC and JS with AMOS 23.0. Preacher and Hayes (2008) put forward that bootstrapping is a type of statistical re-sampling method which estimates the parameters of a model and their standard errors strictly utilizing the sample. Preacher and Hayes (2008) also highlight that bootstrapping computes accurate confidence intervals (CI) of indirect effects when the sampling distribution is not normal. This is closely related to indirect effects because these have distributions that are skewed away from zero (Shrout and Bolger, 2002). In this study, new samples (with replacement) were extracted from our sample 2,000 times to calculate the indirect effect of PsyCap on TI. In line with our expectations, the results of bootstrap confirmed the indirect effect of PsyCap on TI through OC (estimate = −0.151, lower CI = −0.271, upper CI = −0.067, *p* < 0.01) and also through JS (estimate = −0.201, lower CI = −0.467, upper CI = −0.070, *p* < 0.01). Therefore, Hypotheses 6 and 7 can be supported.

## Discussion and Conclusion

### Discussion

PsyCap acted as a predictor of the variables in this study, with direct and indirect effect on outcome. Moreover, PsyCap had a relatively weaker indirect effect on turnover intention through OC than JS. These results imply that turnover intention relies more on JS than OC, which has not been considered before. Hence, current research offers new and interesting findings about the structural role of job attitudes as they relates to PsyCap and turnover intention.

Consistent with the tenets of COR theory (Hobfoll, 1989), four components of PsyCap jointly facilitate and maintain hotel customer-contact employees’ positive job attitudes in current study. Hence, our findings corroborate previous studies on PsyCap and positive attitudes (e.g., Siu et al., 2015; Idris and Manganaro, 2017).

In accordance with Karatepe and Karadas’s (2014) results, our study revealed a negative correlation between PsyCap and turnover intention, in contrast with Abbas et al.’s (2014) research which could not identify such a relationship. What’s more, echoing the research of Paek et al. (2015), and similar to other hotel sector studies (e.g., Bouzari and Karatepe, 2017; Kang et al., 2018), our findings also support indirect associations between PsyCap and turnover intention with two job attitudes as mediators.

Finally, current study examined the relationship of JS (both intrinsic and extrinsic) and OC (affective, continuance and normative) and turnover intention. Many previous studies which have claimed significantly negative association between them (e.g., Tongchaiprasit and Ariyabuddhiphongs, 2016; Jang and Kandampully, 2018; Chen and Wang, 2019), have measured JS or OC as a single construct without dimensions, however, we measured both the extrinsic and intrinsic traits for JS and affective, normative and continuance traits for OC in current research.

### Theoretical Implications

A sample of 228 respondents from eight five-star hotels employed as full-time customer-contact employees (e.g., front desk agents, waiters and waitresses, guest relations representatives, concierges) in Kuala Lumpur was utilized to examine the causal relationships between PsyCap, OC, JS and turnover. The results revealed a good fit between the data and the hypothesized model and further pointed out that PsyCap was significantly and positively associated with OC and JS but negatively correlated with turnover intention. OC and JS were also significantly and negatively correlated with turnover intention. More importantly, OC and JS play important partial mediating roles in the relationships between PsyCap and turnover intention. The causal mediated correlations in this model can be explained via Conservation of Resources Theory (Hobfoll, 1989). Although our findings regarding the effect of PsyCap are similar to the results of some previous studies (e.g., Da et al., 2020; Hua, 2020; Junaid et al., 2020; Sepeng et al., 2020), we provide a new insight in this study that OC and JS exert mediating effects simultaneously. This filled a research gap and declared the effects of OC and JS on turnover intention within a hotel. Furthermore, the results also imply that hotel customer-contact employees who possess high level of PsyCap and positive job attitudes have tendency to stay in their organizations as a consequence. In spite of the plethora of evidence, our research challenges conventional norms, particularly within Malaysian hospitality research landscape.

### Practical Implications

This empirical investigation has several important implications of for hotel management. Firstly, higher level of PsyCap is directly associated with higher JS and OC, and lower turnover intention. In addition, it indirectly relates to lower turnover intention via JS and OC. Thus, hotel management are supposed to realize the importance of PsyCap and evaluate candidates’ PsyCap during selection process. With the availability of psychometrical measurement tools such as PsyCap 24-item questionnaire proposed by Luthans et al. (2007a), hotels can select those candidates who have adequate personal resources. This means that managers can hire right employees with right personal resources for right positions. What’s more, hotel management may consider the evaluation of PsyCap to found out those employees who can succeed in challenging and tough environment.

Second, management can capitalize on appropriate strategies to develop employees’ psychological resources in order to advance their positive organizational attitudes and behaviors (Karatepe and Karadas, 2014). In the first place, hotel management should realize the significance of PsyCap and proactively develop relating training programs to maintain frontline employees’ PsyCap at a high level. Through training programs, employees can learn how to protect, maintain and accumulate their PsyCap more effectively. Besides, more comprehensive training programs which cover employees’ PsyCap and positive job attitudes will be more effective. Therefore, employees in hotels who have received these trainings can master how to avoid the loss of resources. If they are able to maintain high level of psychological resources, they will be highly satisfied with and more committed to their jobs. Simultaneously, they may also feel a strong sense of belonging with their current jobs and have less intention to withdraw from their organizations.

### Limitations

Although this study has made some important contributions to existing knowledge, we must admit that there are still some limitations that can provide a feasible outlook for future research.

We utilize sequential design which has been considered as a preferred alternative to the cross-sectional design for evaluating mediation in current study. As a matter of fact, sequential design is a compromise between the cross-sectional and longitudinal designs since it adds time into the model but has only one measurement for each of predictors, mediators and criterion variables (Mitchell and Maxwell, 2013). According to Maxwell and Cole (2007), a systematic review showed that 77 of 90 empirical studies involving mediation chose to collect cross-sectional data and utilized the approach of data analysis developed by Baron and Kenny (1986). However, Maxwell et al. (2011) found when the actual mediation processes correspond to a longitudinal autoregressive model, cross-sectional designs tend to generate badly biased estimates of direct and indirect effects. In recent years, researchers begin to emphasize temporal precedence in studies of mediation and articles have been published that clearly examine the effect of time sequencing in mediation studies (e.g., MacCallum and Austin, 2000; Maxwell and Cole, 2007; Maxwell et al., 2011). Nevertheless, Maxwell et al. (2011) have identified that sequential designs also provide poor estimates of longitudinal mediation parameters. But sequential designs have an advantage over cross-sectional designs in estimating indirect effect under partial mediation. That is, the direction of the bias in sequential designs is generally more predictable than that in cross-sectional designs (Maxwell et al., 2011). Thus, Maxwell and Cole (2007) have repeatedly highlighted that it’s necessary to take advantage of longitudinal designs when evaluating longitudinal mediation since cross-sectional designs and sequential designs of the MacArthur approach may exaggerate or underestimate direct and indirect effects. In future studies, Mitchell and Maxwell (2013) suggest that researchers should not limit their attention to those longitudinal models which they have referred to in their study. Instead, they are strongly encouraged to consider many other longitudinal autoregressive designs depicted by such sources as Shrout (2011) and Coman et al. (2017) for studying mediation. To some extent, with the improvement of methodology, the nature of psychological processes will be more accurately and clearly revealed (Mitchell and Maxwell, 2013).

### Directions for Future Studies

This study measured TI. If possible, in future studies receiving hotel records regarding actual turnover from work would generate an objective evaluation of employees’ turnover issue. In addition, more antecedents and consequences of PsyCap among customer-contact employees in hotel industry can be verified and the potential relationships can be developed from various theoretical perspectives (e.g., Conservation of Resources Theory, Self-determination Theory, and Job Demands-Resources Model) in future research. This can enhance the comprehensive understanding of PsyCap.

Data were gathered only in five-star hotels in Kuala Lumpur. To some extent this practice may limit the generalizability and representative of the findings. In future research, sampling can be conducted in more regions of Malaysia or in other countries. What’s more, the sample is confined to customer-contact employees working in five-star hotels. Accordingly, future studies are supposed to conduct comparative analysis of different levels of hotels or other sectors in hospitality industry.

### Conclusion

PsyCap is an emerging construct that is attracting more and more attention gradually. Because the research of PsyCap is still scarce in the hospitality field especially in Malaysia, we propose a hypothesized model that integrates PsyCap, JS, OC and turnover intention based on the Conservation of Resources Theory. We obtained empirical evidence to support hypotheses concerning the relationship between these variables mentioned above. The findings reveal that JS and OC partially mediate the relationship between PsyCap and turnover intention. The findings also highlight that PsyCap and job attitudes have critical roles in mitigating turnover intention in the hotel domain. In short, the hypothesized model provides the stage for future studies and provides a valuable reference for future research on PsyCap and turnover intention.

## Data Availability Statement

The raw data supporting the conclusions of this article will be made available by the authors, without undue reservation.

## Ethics Statement

The studies involving human participants were reviewed and approved by University Putra Malaysia Ethics Committee. The patients/participants provided their written informed consent to participate in this study. Written informed consent was obtained from the individual(s) for the publication of any potentially identifiable images or data included in this article.

## Author Contributions

All authors listed have made a substantial, direct and intellectual contribution to the work, and approved it for publication.

## Conflict of Interest

The authors declare that the research was conducted in the absence of any commercial or financial relationships that could be construed as a potential conflict of interest.
